# The clinical and urodynamic outcomes of single-incision mesh surgery using the Uphold system for the treatment of pelvic organ prolapse

**DOI:** 10.1038/s41598-020-69886-z

**Published:** 2020-08-11

**Authors:** Pei-Chi Wu, Chin-Hu Wu, Yiyin Liu, Zixi Loo, Kun-Ling Lin, Cheng-Yu Long

**Affiliations:** 1 Department of Obstetrics and Gynecology, National Taiwan University Hospital, Taipei, Taiwan; 2 Department of Obstetrics and Gynecology, Kaohsiung Medical University Hospital, Kaohsiung Medical University, Kaohsiung, Taiwan; 3 Department of Obstetrics and Gynecology, Kaohsiung Municipal Siaogang Hospital, Kaohsiung Medical University, No. 482, Shan-Ming Road, Hsiao-Kang Dist., Kaohsiung, 812 Taiwan; 4 Department of Obstetrics and Gynecology, Kaohsiung Municipal Ta-Tung Hospital, Kaohsiung Medical University, Kaohsiung, Taiwan

**Keywords:** Quality of life, Urinary incontinence, Outcomes research

## Abstract

This retrospective cohort study aims to assess the clinical and urodynamic outcomes of single-incision mesh surgery with the Uphold system. The medical records of 140 women with anterior and/or apical compartment prolapse stage 2 or greater who underwent Uphold mesh surgeries were reviewed. The clinical evaluation included a pelvic examination, a urodynamic study (UDS), and a personal interview to evaluate lower urinary tract symptoms (LUTS). After a follow-up time of 12–30 months, the anatomical reduction rates were 100% and 96.4% for the apical and anterior compartments, respectively, and these rates were similar across women with or without hysterectomy. All of the LUTS and several UDS parameters improved significantly. The continence rate in women with stress urinary incontinence (SUI) was improved if they also underwent a mid-urethral sling (MUS) operation. However, the continence rate did not differ between women with and without occult urodynamic stress incontinence who did not undergo a concomitant MUS operation. The rate of vaginal mesh extrusion was 2.8%, and this complication rarely occurred beyond the learning curve. In conclusion, the anatomic correction of the Uphold system was satisfactory with a low rate of mesh extrusion. Women with SUI would benefit from a concomitant MUS operation.

## Introduction

Apical prolapse is an important issue among pelvic organ prolapse (POP)^1^, and level 1 support (according to DeLancey’s description) should be provided during repair^2^. In native tissue reconstruction, the sacrospinous ligament is an important attachment point of apical compartment repair. With direct visualization, sacrospinous ligament perforation and retrieval can be aided with specific devices, such as Deschamps ligature carrier^3^, but it is not always easy to approach the ligament from the vagina. The anatomic outcomes of sacrospinous ligament fixation, which is usually performed unilaterally, are heterogenous^4^. Additionally, a higher rate of apical compartment recurrence was reported in sacrospinous hysteropexy than in hysterectomy^5^. Therefore, a more durable procedure suitable for either uterine preservation or hysterectomy is needed^6^, and various transvaginal mesh (TVM) kits have been developed in recent decades.

TVM was once considered a breakthrough innovation in treating POP. Regardless of fair anatomic outcomes, reports about mesh extrusion raised concerns about its safety^7^. Due to the lack of long-term evidence in safety, the US Food and Drug Administration (FDA) ordered all manufacturers to stop selling synthetic mesh intended for transvaginal repair of anterior compartment prolapse on April 16th, 2019^8^. Although TVM surgery is performed less often since the order, a continuous follow-up of women who underwent TVM surgeries is necessary and can provide abundant information. Although numerous procedures regarding level 1 support have been described, fixation with grafts is still one of the options in the flowchart^9^. It appears that TVM still plays a role in POP surgery. Single-incision mesh surgery with the Uphold system was one of the most commonly performed TVM surgeries before the FDA order. The design of the Uphold system is based on bilateral sacrospinous fixation with the aid of a Capio suture capturing device for the apical compartment and a lightweight mesh body covering the anterior compartment.

Since restrictions and concerns regarding TVM increase daily, we need more evidence regarding the efficacy and long-term adverse effects of this procedure compared to native tissue reconstruction, which is usually recognized as having a higher failure rate and requires reoperation for symptomatic recurrence. Our study aimed to provide follow-up data regarding the efficacy, safety, and improvement in patients’ symptoms and the changes in their urodynamic parameters after single-incision mesh surgery with the Uphold system. In addition, de novo stress urinary incontinence (SUI) after Uphold mesh surgery was noticed in a previous study^10^. Our data also provided information about the performance of a concurrent mid-urethral sling (MUS) operation for overt SUI or occult urodynamic stress incontinence (USI).

## Results

Of the 140 women undergoing single-incision mesh surgery for their POP, 135 (96%) had successful anatomic correction in both the apical and anterior compartments, with 4 (2.8%) cases of vaginal mesh extrusion at the 12–30 month follow-up.

The baseline demographic data of the enrolled 140 women were obtained, including age, parity, body mass index (BMI), menopausal status, smoking status, underlying disease, concomitant procedures and follow-up period, as shown in Table 1. The mean age of our study population was 66.0 years old, and the mean parity was 3. The majority of the women (97%) were menopausal, and approximately one-quarter of them had ever undergone a hysterectomy. Among them, 20 women needed posterior repair for their rectocele, and one-third underwent a concomitant MUS operation for overt SUI or occult USI.

**Table 1:** Demographic characteristics of women who underwent single-incision mesh surgery for the treatment of pelvic organ prolapse (n = 140).

Parameters	Mean ± SD
	n (%)
Age (years)	66.0 ± 9.9
Parity	3.0 ± 0.7
BMI (kg/m^2^)	25.2 ± 3.4
Menopause	136 (97.1)
Current smoker	2 (1.4)
Diabetes mellitus	29 (20.7)
Hypertension	70 (50.0)
History of hysterectomy	32 (22.9)
Concomitant procedures in this study	
Posterior repair with mesh	2 (1.4)
Posterior colporrhaphy	18 (12.9)
Vaginal hysterectomy	31 (22.1)
Mid-urethral sling	47 (33.6)
Follow-up (months)	12–30

Data are given as the mean ± standard deviation or n (%).

BMI, body mass index.

### The surgical outcome of POP

All of the POP quantification (POP-Q) measurements improved significantly after the surgeries (Table 2). Five (3.6%) stage 2 cystocele recurrences were documented during follow-up, and all 5 women had stage 3 or 4 apical and anterior prolapse without SUI originally. Mesh extrusion and mesh migration were found in one of these women. Four of the five recurrent women received conservative treatment and regular follow-up due to limited symptoms, and only one woman underwent a second TVM surgery for anterior repair.

**Table 2:** Pelvic organ prolapse quantification (POP-Q) values before and after surgery (n = 140).

POP-Q parameters (cm)	Preoperative	Postoperative	*p* value*
Aa	3 (− 2 to 3)	− 2 (− 3 to 0)	< 0.01
Ba	3 (− 2 to 9)	− 2 (− 3 to 2)	< 0.01
C	3 (− 8 to 10)	− 8 (− 5 to − 13)	< 0.01
Ap	− 2.(− 3 to 3)	− 2 (− 3 to 1)	< 0.01
Bp	− 1 (− 3 to 9)	− 2 (− 3 to 1)	< 0.01
TvL	10 (7 to 13.5)	9.5 (6 to 14)	0.02
Recurrent POP		5 (3.6)	

Data are given as the median (range) or n (%).

TvL, total vaginal length; POP, pelvic organ prolapse.

*The Wilcoxon signed-rank test.

### The improvement over symptoms and changes in urodynamic parameters

In the subjective assessment of patients’ lower urinary tract symptoms (LUTS), all of the symptoms improved significantly after the single incision mesh surgeries, including urinary frequency (64% vs 14%, *p* < 0.01), SUI (49% vs 16%, *p* < 0.01), urgency urinary incontinence (49% vs 6%, *p* < 0.01), feeling of incomplete emptying (84% vs 6%, *p* < 0.01), hesitancy (74% vs 8%, *p* < 0.01), and nocturia (73% vs 41%, *p* < 0.01) (Table 3).

**Table 3:** Urinary symptoms before and 6 months after surgery (n = 140).

Symptoms	Preoperative	Postoperative	*p* value*
Urinary frequency	189 (63.6)	19 (13.6)	< 0.01
Stress urinary incontinence	168 (48.6)	23 (16.4)	< 0.01
Urgency urinary incontinence	169 (49.3)	18 (5.7)	< 0.01
Feeling of incomplete bladder emptying	118 (84.3)	18 (5.7)	< 0.01
Hesitancy	103 (73.6)	11 (7.9)	< 0.01
Nocturia	102 (72.9)	57 (40.7)	< 0.01

Data are given as n (%).

*McNemar’s test.

The postoperative continence rate in women who underwent single-incision mesh surgery is shown in Table 4. For 68 overt SUI women, the continence rates in mesh surgery in women who had undergone and who had not undergone a MUS operation were 89.2% and 67.7%, respectively. On the other hand, 72 women did not complain of SUI before the operation. Ten of them underwent a concomitant MUS operation due to a history of SUI and/or occult USI, and the continence rates were 90%. For the remaining 62 continent participants who underwent solitary mesh surgery, the continence rates were 85% in women with occult USI and 88% in women without occult USI.

**Table 4:** Comparison of postoperative continence rate in women with and without preoperative stress urinary incontinence.

Preoperative symptom	Surgery	Continence rate (%)
SUI (n = 68)	Uphold + MUS (n = 37)	89.2
	Uphold (n = 31)	67.7
No SUI (n = 72)	Uphold + MUS (History of SUI and/or occult USI, n = 10)	90
	Uphold (Occult USI, n = 13)	84.6
	Uphold (No occult USI, n = 49)	87.8

SUI, stress urinary incontinence; MUS, mid-urethral sling; USI, urodynamic stress incontinence.

The objective evaluation of lower urinary tract function with urodynamic study (UDS) revealed significant changes in some parameters after the surgeries, including a decrease in detrusor overactivity (DO) (33% vs 18%, *p* = 0.02), a decrease in residual urine (RU) (77.3 ± 35.2 vs 42.6 ± 21.9, *p* < 0.01), a decrease in detrusor pressure at peak flow (PdetQmax) (49.5 ± 30.6 vs 32.1 ± 13.5, *p* < 0.01), an increase in functional urethral length (FUL) (24.4 ± 8.5 vs 25.7 ± 6.3, *p* = 0.05), and a decrease in maximum urethral closure pressure (MUCP) (58.6 ± 32.3 vs 46.3 ± 12.4, *p* < 0.01) (Table 5).

**Table 5:** Urodynamic changes before and 6 months after surgery (n = 140).

Parameters	Preoperative	Postoperative	*p* value
Qmax (mL/s)	16.3 ± 5.8	17.3 ± 8.2	0.37**
RU (mL)	77.3 ± 35.2	42.6 ± 21.9	< 0.01**^#^
DO	46 (32.9)	25 (17.9)	0.02*^#^
FS (mL)	175.5 ± 85.4	183.0 ± 54.6	0.33**
MCC (mL)	370.8 ± 164.5	349.7 ± 93.0	0.15**
PdetQmax (cmH_2_O)	49.5 ± 30.6	32.1 ± 13.5	< 0.01**^#^
FUL (mm)	24.4 ± 8.5	25.7 ± 6.3	0.05**^#^
MUCP (cmH_2_O)	58.6 ± 32.3	46.3 ± 12.4	< 0.01**^#^
UCA (mmcmH_2_O)	756.8 ± 362.3	707.7 ± 285.0	0.06**

Data are given as the mean ± standard deviation or n (%).

Qmax, maximum flow rate; RU, residual urine; DO, detrusor overactivity; FS, first sensation to void; MCC, maximum cystometric capacity; PdetQmax, detrusor pressure at peak flow; FUL, functional urethral length; MUCP, maximum urethral closure pressure; UCA, urethral closure area.

*Chi-square test.

**The paired *t*-test.

^#^Statistical significance.

### Intraoperative and postoperative complications

No intraoperative complications were noted in the study period (Table 6). Postoperative surgical wound pain was tolerable. Nineteen (14%) of the 140 participants experienced urinary tract infection after surgery, and all of them were treated with oral or intravenous antibiotics for 3 to 7 days without any sequelae. Fifteen (11%) women had voiding difficulty after mesh surgery (9 with concomitant MUS and 6 without MUS), but all of the symptoms resolved after conservative treatment. One (1%) woman had perineal hematoma that resolved completely at her 1-month follow-up. Four (2.8%) vaginal mesh extrusions were found during the follow-up period, and one (1%) of woman needed surgical management and was classified as grade III in the Clavien–Dindo classification (Table 7).

**Table 6:** Intraoperative, postoperative and mesh-related complications (n = 140).

Complication	n (%)
Intraoperative complications	
Bladder injury	0
Rectal injury	0
Blood transfusion	0
Postoperative complications	
Urinary tract infection	19 (13.6)
Voiding difficulty	15 (10.7)
Perineal hematoma	1 (0.7)
Mesh complications	
Vaginal extrusion	4 (2.8)
Bladder extrusion	0

Data are given as n (%).

**Table 7:** The grading of complications according to Clavien–Dindo classification (n = 140).

Clavien–Dindo Classification	UTI	VD	Perineal hematoma	Vaginal mesh extrusion
Grade I			1 (0.7)	
Grade II	19 (13.6)	15 (10.7)		3 (2.1)
Grade III				1 (0.7)
Grade IV				
Total	19 (13.6)	15 (10.7)	1 (0.7)	4 (2.8)

Data are given as n (%).

UTI, urinary tract infection; VD, voiding difficulty.

## Discussion

For women with dominant apical prolapse, single-incision mesh surgery using the Uphold TVM system resulted in an ideal anatomical reduction and an improvement in LUTS, with an acceptable vaginal mesh extrusion rate.

TVM surgeries had once thrived for decades due to favorable surgical outcomes and an easy approach; they evolved from transobturator 4-arm mesh surgery^11^ to single-incision mesh surgery^12^. However, public concerns regarding the safety of TVM never subsided. On April 16, 2019, the FDA ordered all manufacturers to stop selling synthetic mesh intended for transvaginal repair of anterior compartment prolapse because the manufacturers have not demonstrated a reasonable assurance of safety and effectiveness for these devices^8^. On the other hand, sacrocolpopexy or sacrohysteropexy serves as the gold standard for the treatment of apical prolapse due to its high efficacy and lower risk of adverse events, such as dyspareunia^13^. However, the benefits should be balanced against the technical threshold of laparoscopic sacrocolpopexy, which would decrease the accessibility of this surgery^14^. Although the Uphold system was not allowed to be produced since the FDA announcement, it is still important to follow up with the women who underwent pelvic reconstruction surgery with single-incision mesh.

Our data revealed that the anatomical reduction rate was 96.4% in the anterior compartment and 100% in the apical compartment. Among the 5 women with anterior compartment recurrence, one underwent a concomitant hysterectomy, and two had previously undergone a hysterectomy. One patient underwent reoperation (1/140, 0.7%) for symptomatic cystocele. The surgical efficacies of anterior compartment prolapse were 96.8% and 96.3% in single-incision mesh surgery with the Uphold system with and without hysterectomy, respectively. These results were comparable to previous studies, in which the reduction rates ranged from 84 to 97.2% in the apical compartment^6,10,15–21^ and 77.6% to 97% in the anterior compartment^6,16–19,21,22^. This outcome was more similar between different studies compared to the heterogeneous outcome of sacrospinous ligament fixation^4^. A prospective study revealed that a high-volume surgeon would have a better anatomical result^17^. Hsieh et al. defined more specifically that anatomic failure after a certain single-incision vaginal mesh procedure was significantly associated with the initial 35 cases^23^, indicating the obvious learning curve in pelvic reconstruction surgery with mesh. The two operators in our study are both high-volume urogynecologists, and this would have a great impact on the success rate of single-incision mesh surgery with the Uphold system. However, we did not find a specific case number after which the failure rate declined dramatically. The anterior vaginal mesh may not be available anymore, but the design of apical fixation using the Uphold system with durable outcomes can still serve as an idea for the development of equipment.

The Uphold system can be applied either with hysterectomy or with uterine conservation. We previously evaluated the Uphold system’s performance among women with or without concomitant hysterectomy and found comparable outcomes in both groups^6^. On the basis of this study, uterine preservative pelvic reconstruction surgery is much more performed than hysterectomy in our clinical practice, and in this study, the data revealed consistency regarding the issue of uterine sparing or hysterectomy.

It has been recognized that many LUTS related to cystocele would be improved after pelvic reconstruction surgery^24,25^ regardless of whether the mesh was used^6^ or other pelvic reconstruction methods were used^12,26^. We observed consistent findings in the improvement of LUTS in both voiding functions and storage functions, such as urgency urinary incontinence (UUI). Women with UUI or overactive bladder (OAB)-wet are usually treated with antimuscarinic agents instead of operation^27^. However, based on integral theory, once the structure or ligaments of women with LUTS are repaired, the function will be restored^28^. There are correlations between the improvement of subjective LUTS and changes in objective UDS parameters. After correcting the cystocele, the women could maintain a similar flow rate with a lower detrusor pressure, resulting in less residual urine, which decreases the likelihood that the women would complain of incomplete bladder emptying. The DO was also decreased significantly, indicating that fewer of the women would suffer from urinary frequency, urgency, and nocturia, which were supposed to be managed with medication. The restored apex may straighten the urethra, resulting in a longer FUL and lower MUCP, but the MCUP did not decrease so much that it led to incontinence. These findings correlated with the integral theory of pelvic floor.

De novo SUI is a major issue in pelvic reconstruction surgery. Compared to native tissue repair, armed anterior vaginal mesh repair may slightly increase the likelihood of de novo SUI^29^. Different mesh designs would result in different predictors of de novo SUI^30,31^, and Jelovsek and his colleagues developed a model to predict the likelihood of de novo SUI after POP surgery^32^, which may facilitate decision making regarding concomitant MUS. However, due to the relatively low incidence of de novo SUI, Alas suggested that the patients should be counseled about the risk and offered a staged procedure if needed^33^. In addition, concomitant MUS may make it more difficult to determine the cause of postoperative voiding dysfunction. For women with overt SUI, occult USI, or previous SUI history^30^, the option of concomitant MUS or staged surgery was offered in our study, and they made the decision based on the shared decision-making principle. For women with overt SUI, concomitant MUS largely improved the postoperative continence rate, which is comparable to a previous study^29^. For women without SUI, the postoperative continence rate did not differ significantly between the with and without occult USI groups. Among them, women with a history of SUI may benefit slightly compared to women without a history of SUI. Therefore, only women with POP and SUI are appropriate candidates for mesh surgery with the Uphold system and concomitant MUS.

Postoperative voiding difficulty occurred more frequently in women who underwent a concomitant MUS operation (9/47, 19%, five were occult USI and 4 were overt SUI) than women who did not (6/93, 6%). Therefore, staged operation is a reasonable choice for women with POP and occult USI, and this option should also be provided to women with SUI. In addition, mild to moderate SUI can also be managed with conservative and noninvasive treatment, such as Kegel exercises, vaginal laser, bulky agent injection, or some novel treatments (e.g., intensity extracorporeal low energy shock wave therapy)^34^.

No intraoperative complications were noted during the study period. The most common postoperative complication was urinary tract infection, which can be managed with oral or intravenous antibiotics. No chronic infection was noted during the follow-up period. Regarding voiding difficulty, most of the women with postoperative voiding difficult resolved a few days later. Only one patient needed to receive clean intermittent urine catheterization, and the symptom resolved within 1 week. There was no bladder mesh extrusion, but four women (2.8%) had vaginal extrusion, and one of them underwent local mesh excision. None of the women with mesh extrusion underwent concurrent hysterectomy. The mesh extrusion rate was comparable to previous studies using the Uphold system for POP surgeries, ranging from 0 to 6.6%^6,10,15–22^. There was no mesh extrusion after the 52nd surgery, which may indicate that there was a learning curve for the Uphold mesh surgery.

Compared to previous market mesh kits^7^, the surgical outcome can considerably outweigh the adverse events. These results may have occurred because the lightweight type 1 mesh body of the Uphold system reduced the possibility of mesh folding-related poor tissue remodeling. Additionally, Lo illustrated that the Uphold system has no anterior arm over the apex of the vaginal vault, thus decreasing the risk of hysterectomy-related mesh extrusion at the apex^10^. There was no pelvic pain noted in our study during the follow-up period, but a patient suffered from leg pain a few days after the operation and was diagnosed with sciatica by the neurologist.

We acknowledge that the limitations of our study are the retrospective design and the limited follow-up period. For both transvaginal and transabdominal mesh surgery, a 2-year or longer follow-up sometimes cannot reveal all the complications and durability. However, due to a relatively lightweight mesh body and intraoperatively minimal dissection, a very low risk of severe complications, such as bowel perforation and bladder perforation, can be expected in long-term follow-up. Additionally, our study provides additional information by comparing the continence rate of concurrent MUS for women with and without SUI and analyzed the relationship between concomitant surgery and postoperative voiding difficulty. In addition, the improvement of all LUTS was discussed and provided more supportive evidence of the integral theory. A study with a longer follow-up of the Uphold system should be conducted to confirm the long-term efficacy of apical fixation.

## Patients and methods

From October 2015 through August 2017, the medical records of women who underwent single-incision mesh surgery with the Uphold system at a tertiary referral center in Taiwan were retrospectively reviewed. Women with stage 2 or greater anterior and/or apical compartment prolapse (as defined by the POP-Q staging system^35^) were included. We excluded women who had incomplete data or were lost to follow-up within 12 months after the surgery. Finally, this cohort study was conducted on the basis of 140 available subjects.

The baseline demographic data included age, parity, body mass index (BMI), menopausal status, smoking condition, underlying disease, hysterectomy history, baseline POP stage using the POP-Q system^36^, concomitant procedures, and follow-up period. The review of the chart records consisted of a detailed history before and after the surgery until the patient’s last follow-up, including urinary analysis, pelvic examination, UDS, transabdominal ultrasound, and personal interview to identify any urinary symptom taking into account the 2002 International Continence Society (ICS) definitions^37^. UDS, including uroflowmetry, filling and voiding cystometry, and urethral pressure profilometry, were performed according to the recommendations by the ICS^38^ with a 6-channel urodynamic monitor (MMS; UD2000, Enschede, Netherlands) by an experienced technician. Any uninhibited detrusor contraction during filling cystometry was deemed positive for DO. The diagnosis of occult USI was made by the occurrence of urinary leakage in the stress test after prolapse reduction with vaginal gauze roll with 250 mL urine in the bladder.

Preoperative counseling pertaining to uterine preservation, surgical procedures, safety, efficacy, and potential complications were given. The option of concomitant MUS operation was given to women suffering from overt SUI, occult USI, or history of SUI. The possibility of postoperative voiding difficulty was well explained, as well. After counseling, the patient chose concomitant hysterectomy and/or MUS in addition to single-incision mesh surgery based on a shared decision-making principle. These MUS procedures included MiniArc (AMS, Inc., Minnetonka, MN, USA) and TVT-O (Gynecare TVT-Obturator System, Ethicon, Inc., Somerville, NJ). Concomitant posterior colporrhaphy was performed as needed.

### Operative technique: the Uphold system

All surgical procedures were performed under general anesthesia by two experienced urogynecologists in accordance with the original advice of the Uphold system. Every patient received a single dose of intravenous prophylactic antibiotics. The patients were placed in a lithotomy position. After single urine catheterization and hydrodissection with diluted vasopressin (Pitressin, Parke Davis Division, Warner Lamber Canada Inc., Canada), a vertical incision was made over the anterior vaginal wall, from the bladder neck to the most descent part of the prolapse. Full thickness dissection was performed to separate the endopelvic fascia and anterior vaginal mucosa. The operator kept dissecting the paravesicle space until reaching the bilateral sacrospinous ligaments. Dissection with one- to two-finger breadths further down from ischial spines towards the sacrum was performed. The mesh arms of the Uphold LITE Vaginal Support System (Boston Scientific, Marlborough, MA, US) were attached to the bilateral sacrospinous ligaments with the aid of the Capio suture capturing device. The mesh was attached to native vaginal tissue with PDS 2-0 (Ethicon, NJ, US), followed by anterior colporrhaphy using Vicryl 2-0 (Ethicon, NJ, US). The mesh arms were pulled simultaneously at the same pace until the prolapse was reduced to its natural position. Then, we performed a cystoscopy to check whether there was any bladder or ureter injury. The excessive mesh arms were removed. The vaginal wound was closed with Vicryl 2-0 sutures. A roll of vaginal gauze for compression and a Foley catheter were applied for 48 h. If concurrent surgeries were scheduled to perform, vaginal hysterectomy was performed before mesh insertion, and the MUS operation and posterior colporrhaphy were performed after mesh insertion.

As a follow-up, postoperative outpatient visits were at 1, 2, 3, 6, and 12 months and then semiannually beyond 1 year. Pelvic examination was performed routinely at every visit to clinics. The postoperative UDS was performed at 6 months after the surgery, and LUTS were assessed at the same visit. The primary outcome of this study was the anatomical reduction rate after surgery, assessed objectively by pelvic examination using the POP-Q system. Recurrence was defined as POP-Q stage 2 or more found at any follow-up visit. Secondary outcomes included assessment of subjective LUTS, objective UDS performance, intra- and postoperative complications, and continence rate. The Clavien–Dindo classification was used for the grading of the intraoperative and postoperative complications^39^. Postoperative voiding difficulty was defined as an RU greater than 100 mL under sonography after three micturitions.

### Statistics

IBM SPSS Statistical Software version 20.0 ed. was used for statistical analyses. The Wilcoxon signed-rank test was performed for comparisons between preoperative and postoperative POP-Q parameters. McNemar's and Chi-square tests were performed for categorical variables. The paired *t*-test was performed for two related units on a continuous outcome. A *p* value of less than 0.05 was considered statistically significant.

### Ethical approval and ClinicalTrials.gov registration

This study received approval from the Institutional Review Board of Kaohsiung Medical University Hospital (ID: KMUHIRB-E(I)-20190015), by which relevant guidelines and regulations were followed accordingly. This study was also registered at ClinicalTrials.gov (ID: NCT04139083, registered on October 24th, 2019).

### Informed consent

Informed consent was obtained from all participants before surgery.

## Conclusions

Single-incision mesh surgery with the Uphold system provides not only a durable anatomic reduction rate, especially in the apical compartment, but also an improvement over all kinds of LUTS among women with or without hysterectomy. For women with POP and SUI, a concurrent MUS operation can lead to a better postoperative continence rate. Staged operation is a reasonable choice for women with POP and occult USI because of the increasing possibility of voiding difficulty. A low risk of mesh extrusion was noted, and this complication rarely occurred beyond the learning curve. This information is useful for preoperative counseling for POP surgery.

## Data Availability

The datasets analyzed during the current study are available from the corresponding author on reasonable request.
